# Low influenza vaccine uptake by healthcare workers caring for the elderly in South African old age homes and primary healthcare facilities

**DOI:** 10.1186/s12889-022-14926-8

**Published:** 2023-01-12

**Authors:** Mncengeli Sibanda, Johanna C. Meyer, Brian Godman, Rosemary J. Burnett

**Affiliations:** 1 Department of Public Health Pharmacy and Management, School of Pharmacy, Sefako Makgatho Health Sciences University, Ga-Rankuwa, Pretoria, South Africa; 2 South African Vaccination and Immunisation Centre, Sefako Makgatho Health Sciences University, Ga-Rankuwa, Pretoria, South Africa; 3 Department of Pharmacoepidemiology, Strathclyde Institute of Pharmacy and Biomedical Sciences, University of Strathclyde, Glasgow, UK; 4 Centre of Medical and Bio-allied Health Sciences Research, Ajman University, Ajman, United Arab Emirates; 5 Department of Virology, School of Medicine, Sefako Makgatho Health Sciences University, Ga-Rankuwa, Pretoria, South Africa

**Keywords:** Influenza, Healthcare workers, Elderly, Vaccine, Vaccination, South Africa

## Abstract

**Background:**

The elderly bear the highest burden of South Africa’s estimated annual > 10 million influenza cases and > 11,000 influenza-related deaths. Unvaccinated healthcare workers (HCWs) are at high occupational risk of contracting influenza, and may transmit influenza to elderly patients in their care. Thus, the South African National Department of Health recommends that HCWs receive annual influenza vaccination. This study aimed to determine influenza vaccination coverage among HCWs; identify reasons for their vaccination status; and investigate if HCWs recommend vaccination to their elderly patients.

**Methods:**

A descriptive study was conducted in 18 community health centres and 44 private sector and non-governmental organisation managed old age homes across South Africa, using a self-administered structured questionnaire, which was distributed to 360 HCWs present on the day of data collection. Data were captured using Microsoft Excel® and imported to Epi Info™ 7 (Centers for Disease Control and Prevention, USA) for descriptive statistical analysis. Ethics approval (SMUREC/P/36/2018: PG) and permission to conduct the study at the facilities were obtained. All participants provided informed consent.

**Results:**

The response rate was 76.7% (276/360). Most participants were female (90.9% [251/276]), nursing professionals (81.2% [224/276]) with a mean age of 41.1 ± 11.7 years. Although 62.7% of participants indicated having ever received at least one dose of the influenza vaccine, influenza vaccine uptake for 2017 and 2018 was 24.36% (41/276) and 33.3% (92/276) respectively. The main reasons given for never being vaccinated against influenza were related to the unavailability of the vaccine (70.9%) and vaccine hesitancy (27.2%). Most participants (67.8% [187/276]) recommended vaccines to elderly patients in their care.

**Conclusion:**

The main reasons behind low influenza vaccine uptake by HCWs in South Africa who care for the elderly were related to unavailability of the vaccine and vaccine hesitancy. Strategies to educate HCWs on the importance of influenza vaccination, while concurrently increasing sustained and easy access to the vaccine by HCWs are needed to preserve public health.

**Supplementary Information:**

The online version contains supplementary material available at 10.1186/s12889-022-14926-8.

## Background

Human influenza is a highly infectious, acute upper respiratory tract infection caused by influenza viruses A, B and C, genera of the *Orthomyxoviridae* family which are enveloped negative-sense single stranded RNA viruses [1, 2]. These viruses have segmented genomes and an error-prone replication process, allowing for gene reassortment (antigenic shift), and mutations (antigenic drift) respectively [1]. Antigenic drift results in seasonal influenza epidemics caused mainly by influenza A virus, and less commonly by influenza B virus. Influenza C virus is uncommon, and is not associated with seasonal influenza epidemics [1, 2]. Besides infecting humans, influenza A virus infects several other species of mammals and birds. Consequently, interspecies gene reassortment can occur, resulting in sporadic global pandemics [1, 2]. Influenza B virus infects mainly humans and has also been found in seals. However, while interspecies reassortment is possible, it is unlikely, which is probably why influenza B virus does not cause pandemics [2].

Influenza viruses are transmitted person-to-person via respiratory droplets and aerosols, and touching contaminated fomites [2–4]. Healthcare workers (HCWs) are at increased risk of contracting influenza infection due to their frequent exposure to infected patients [5–8]. In addition, infected HCWs may pose a potential risk of further transmitting influenza to patients in their care, particularly high-risk patients such as the elderly, those with impaired immunity and those with comorbidities [5–7]. Studies have shown that HCWs often continue to work despite being infected with influenza [6–10]. The reasons cited include an obligation to their patients and other colleagues despite their illness, as well as sick leave restrictions [11, 12].

Generally, influenza is a mild and self-limiting illness. However, it may develop into severe illness and result in death, depending on the influenza strain, and patient factors including age, immune status and comorbidities such as chronic pulmonary or cardiovascular, renal, hepatic, neurologic, hematologic, or metabolic disorders (including diabetes mellitus) [13]. The elderly (persons aged ≥60 years) are at high risk of influenza and its complications mainly due to immunosenescence (ageing-related gradual deterioration of the immune system, with reduced innate and adaptive immune system responsiveness) [14–16]. Globally, influenza causes significant morbidity and mortality, with an estimated one billion cases and 291,243 to 645,832 deaths annually during 1999-2015, with the highest death rates occurring amongst the elderly [17, 18]. In South Africa, prior to the introduction of non-pharmaceutical interventions in 2020 to mitigate the coronavirus disease 2019 (COVID-19) pandemic, influenza was estimated to cause over 128,000 episodes of severe influenza-related illness and over 11,000 deaths every year, with almost 40% of these deaths occurring in the elderly [19].

Seasonal influenza vaccination is a safe and cost-effective strategy for prevention of influenza and its related complications [1, 4, 13, 20, 21]. Generally, HCWs are usually healthy and not at high risk of influenza complications when compared to the general population or patients under their care [22]. Nevertheless, vaccination of HCWs should still be considered an important part of infection control strategies in any healthcare facility, since vaccination provides indirect protection to their unvaccinated patients through herd immunity [5–7, 12]. Several studies have found that increased influenza vaccination in HCWs is associated with decreased nosocomial transmission of influenza, and consequently lower rates of influenza-like illnesses and death amongst patients [5–7, 12, 23]. While a systematic review of influenza vaccination of HCWs who care for the elderly living in long-term care institutions found inconclusive evidence of the benefits for the elderly, this lack of evidence was due to the very low quality of studies and a lack of data [24]. It is also important to vaccinate HCWs in order to reduce absenteeism due to influenza-related illnesses thereby ensuring that healthcare services are maintained with sufficient staff, especially during the COVID-19 pandemic and influenza epidemics [25, 26]. In recognition of these benefits, many countries have policies and guidelines for HCW influenza vaccination programmes [5, 6, 21, 25, 27–31]. Despite these policies and guidelines, influenza vaccine coverage rates and acceptance amongst HCWs were found to be low in many countries [25, 32–35].

The South African National Department of Health (NDoH), the National Institute for Communicable Diseases, the South African Thoracic Society and the Federation of Infectious Diseases Societies of Southern Africa, have published guidelines which highly recommend influenza vaccination for HCWs [31, 36–38]. However, prior to 2020, the NDoH provided free influenza vaccination only to HCWs at high risk of severe influenza outcomes (pregnancy; aged >65 years; underlying medical conditions such as heart disease, lung disease, and human immunodeficiency virus [HIV] infection) [36]. All other HCWs could access the influenza vaccine at their own cost in the private sector. This is despite free and easy access to influenza vaccine being associated with a higher likelihood of getting vaccinated [39].

A study conducted on HCWs in five South African provinces in 2013/14, found an influenza vaccine uptake of only 34% [39]. Similarly, a study conducted amongst HCWs in primary healthcare clinics in the Bojanala District of South Africa found vaccine uptake of only 28.7%. Smaller studies conducted in the Western Cape Province have also reported low uptake, ranging from 25% of pediatric HCWs working at a tertiary hospital [11] to 26.4% of university student HCWs [40]. An even lower uptake of 22.1% was reported in a study conducted at a tertiary hospital in Gauteng Province [41]. However, South African data on influenza vaccine uptake amongst HCWs caring for the elderly are lacking. This is important given the vulnerability of the elderly to influenza.

This study aimed to address this by investigating influenza vaccination practices of HCWs caring for the elderly in South Africa. The objectives of this study included (a) determining current influenza vaccination coverage rates; (b) identifying reasons for accepting influenza vaccination; (c) identifying reasons for not having received influenza vaccination; and (d) investigating if HCWs recommend vaccination to their elderly patients.

## Methods

### Study setting and design

South African community health centres (CHCs) are public sector down-referral health facilities for district hospitals. There are 3,468 CHCs in South Africa and these second-tier healthcare facilities offer primary healthcare to the majority of South African elderly for their preventative and curative healthcare needs [42–44]. South Africa also provides healthcare to the elderly at old age homes (OAHs) [45]. OAHs are facilities used primarily for the purpose of providing long-term assisted living or frail care services 24 hours a day, 7 days a week, to elderly persons who can no longer be cared for in the community due to physical or mental frailty. There is communal sharing of services such as healthcare, meals, activities and entertainment. The South African National Department of Social Development (DSD) maintains a national register of all registered OAHs, which contains information on the location (address, province, magisterial district), contact details and sector (private, public and non-governmental organisation [NGO]). There are currently over 400 OAHs registered with the DSD of which 234 are NGO run OAHs, 170 are independently (privately) run OAHs, and eight are state managed (public) and fully subsidised by the government [46]. The number of facilities per province are shown in Table 1.

**Table 1:** Distribution of health facilities and study sites in the final study sample [42, 43]

Province	CHCs*		OAHs**			
			Private		NGOs	
	Total	Final sample	Total	Final sample	Total	Final sample
**Eastern Cape**	768	2	22	3	27	1
**Free State**	222	2	15	2	18	2
**Gauteng**	370	2	37	4	48	5
**Kwa-Zulu Natal**	605	2	18	2	26	4
**Limpopo**	480	2	1	0	6	3
**Mpumalanga**	288	2	6	1	12	3
**Northern Cape**	161	2	10	1	16	2
**North West**	309	2	14	3	11	2
**Western Cape**	265	2	47	2	70	4
**TOTAL**	3468	18	170	18^#^	234	26^#^

*CHCs* Community health centres, *OAHs* Old age homes, *NGOs* Non-governmental organizations

^#^Total number of OAHs (private and NGO) in final sample: 44

Sources: *https://www.hst.org.za/publications/Pages/DISTRICT-HEALTH-BAROMETER-201819.aspx

**https://social.un.org/ageing-working group/documents/FINAL%20REPORT%20DSD%20Audit%20of%20Residential%20Facilities%20April2010.pdf

A descriptive quantitative study using a self-administered structured questionnaire was conducted amongst HCWs working in selected CHCs and selected public, NGO and private sector OAHs in South Africa. This study was part of a larger study entitled *“Appropriate antimicrobial and vaccine use via mobile health and other techniques in the Republic of South Africa (ENAABLERS project)”* [47] and was approved by the Sefako Makgatho University Research Ethics Committee, (SMUREC/P/36/2018: PG).

### Sampling of study sites

This study was planned to be conducted in 18 CHCs (2 per province) and 27 OAHs (3 per province) in all nine provinces of South Africa (see Fig. S1). The 18 CHCs for this study were the same study sites identified for the ENAABLERS project, the sampling for which was based on provincial academic tertiary hospitals participating in the project, as starting points for downstream selection of one feeder district hospital per province. Details of the sampling methods have been published elsewhere [48]. In summary, the 18 CHCs were selected conveniently, stratified per province, with consideration of the following: being a down-referral facility for the selected district hospital; close proximity to the district hospital; ease of access; financial considerations; and other logistical reasons [48]. OAHs in closest proximity to the selected CHCs, with a minimum capacity of 100 residents (to allow for an adequate sample size), were purposively selected for this study in order to give equal representation in each province of OAHs run by the different sectors, principally concentrating on the private sector and NGO run homes as these were the most prevalent. Where permission was not granted, the next closest OAH with a minimum capacity of 100 residents was approached.

### Study population and sample size

Given the explanatory and descriptive nature of this study, and the fact that participants would come from a multiplicity of geographical locations (all nine provinces in South Africa) as well as different kinds of facilities that specialise in the socio-medical management of the elderly (CHCs and OAHs), a multi-stage sampling method was used. After the first stage (sampling of facilities as described above), second stage sampling was planned to ensure proportional representation of participants per facility (CHCs and OAHs) per province especially among private and NGO OAHs. Based on a study conducted at the same CHCs [47, 48] it was estimated that on average 20 HCWs were employed at each of the 18 CHCs (i.e. 360 HCWs in total). Based on telephonic communication with a sample of OAHs, it was estimated that in general, no more than 10 HCWs were employed at each of the 27 OAHs (i.e. a maximum of 270 HCWs). This gave a total study population of 630 HCWs.

Owing to the special characteristics of the participants (HCWs who care for the elderly), a higher level of influenza vaccine uptake (50%) was assumed when compared with other South African HCWs (the highest being 34% [37]. Using 50% vaccine uptake also gives the largest sample size for conducting population surveys. Based on a study population of 630 and influenza vaccine uptake of 50%, a sample size of 239 was calculated at a 95% confidence level with ±5% margin of error (90% power), using Epi Info™ 7 (Centers for Disease Control and Prevention, USA). The sample size was increased to 360, to allow for non-response and incomplete data. See Fig. S1 for a schematic outline of the study sites and number of HCWs that were planned to be included in the sample.

All HCWs at the study sites, including general medical practitioners, specialist physicians, general and frail care nurses, pharmacists and other allied HCWs were eligible to participate.

### Data collection tool

The self-administered questionnaire was developed based on the literature [27, 49, 50] and in consultation with experts in the field of vaccines to ensure content and face validity. The questionnaire was available in English (the official language for the workplace in South Africa), and included a total of 14 questions. The first section concerned the socio-demographic data of the HCWs such as age, profession, employment setting (OAH or CHC) and sector (for OAHs: NGO, private, public), ethnic group and years of clinical experience. The second section contained questions on influenza vaccine uptake (prior uptake and uptake during the 2018 influenza season); reasons for vaccine acceptance for vaccinated HCWs; reasons for being unvaccinated for unvaccinated HCWs; and whether or not the HCW recommended vaccines to their elderly patients.

The questionnaire was pre-tested on five volunteer HCWs at the out-patient department at an academic hospital in Pretoria, South Africa, for comprehension of instructions, clarity/understanding of questions, time and ease of completion. Volunteers were interviewed after completion of the questionnaire in order to ascertain if the answers they gave were in line with the interpretation of their answers by the researchers. Based upon volunteer feedback after the pre-testing of the questionnaire, some minor corrections were made to the questionnaire before use in the full study. These steps enhanced the robustness and validity of the questionnaire.

### Data collection and recruitment of study participants

Data were collected by MS and a research assistant during weekdays (08h00 to 16h00) from August 2018 to December 2018. Permission to conduct the study at the CHCs and OAHs was obtained from the provincial departments of health and provincial departments of social development respectively, and the facility managers of all CHCs and OAHs. Arrangements were made with facility managers on the most suitable date and time for data collection prior to the actual study visit.

All eligible HCWs present at the facilities on the day of data collection were given a brief introduction to the study, including its aims and objectives, data protection and privacy issues, and were invited to participate. Signed informed consent was obtained from the first 11 (CHCs) or six (OAHs) volunteers, after which the self-administered questionnaire was distributed. Participants were required to complete the questionnaire anonymously (i.e. personal identifiers were not included in the questionnaire), as soon as possible on the same day, and encouraged not to discuss their responses with others to further improve on the reliability of information given. They were also given instructions on how to complete the survey and to place the completed questionnaires in a sealed box that was available at each study site. Sealed boxes were collected by the data collectors at the end of the day.

### Data management and statistical analysis

The collected data were captured using Microsoft Excel® (Microsoft Office, 2016) by the principal researcher (MS) and a research assistant. Double-checking of data entry was undertaken to ensure the reliability of data capture after which the data were cleaned using Microsoft Access® (Microsoft Office, 2016) (i.e. cross-checked for consistency and completeness). Records with contradictory or incomplete data were removed. Continuous data (age and work experience) were collapsed into categorical data. Data were exported to Epi Info™ 7 (Centers for Disease Control and Prevention, USA) for descriptive statistical analysis. This included calculating means with standard deviation (SD) and ranges for continuous variables, and frequency distributions for categorical variables. All frequency data were reported as percentages and a stratified analysis based on vaccine uptake was performed for socio-demographic characteristics of HCWs. Names of facilities were not captured, but were coded and these data were kept confidential.

## Results

### Final sample and response rates

The distribution of the final sample of HCWs from the nine provinces, the types of facilities and sectors, is summarised in Table 2. Of the 373 HCWs present at the 18 CHCs on the days of data collection, 236 accepted to complete the questionnaire, giving an initial response rate of 63.3% for HCWs from CHCs.

**Table 2:** Distribution of HCWs present at the study sites on the day of data collection, and the final sample

Province	HCWs at CHCs		HCWs at OAHs			
			Private		NGOs	
	Present	Final sample	Present	Final sample	Present	Final sample
**Eastern Cape**	51	30	11	9	4	4
**Free State**	40	14	6	5	5	4
**Gauteng**	74	40	16	14	19	16
**Kwa-Zulu Natal**	34	21	6	5	12	10
**Limpopo**	29	33	0	0	6	5
**Mpumalanga**	33	21	4	3	8	6
**Northern Cape**	22	22	3	2	5	4
**North West**	30	20	10	9	7	5
**Western Cape**	60	35	10	9	17	14
**TOTAL**	373	236	66	56^*^	83	68^*^

*CHCs* Community health centres, *OAHs* Old age homes, *NGOs* Non-governmental organizations

^*^Total number of HCWs surveyed at OAHs: 124

Obtaining permission from the eight public sector OAHs was not successful, consequently these OAHs had to be excluded from the study. As a result and to achieve the targeted sample size of 360 HCWs, the number of OAHs that were approached and gave permission to participate had to be increased from 27 to 44 because of insufficient numbers of HCWs employed at the OAHs and low response rates at some of the OAHs. Of the 149 HCWs present at the OAHs on the day of data collection, 124 accepted to complete the questionnaire, giving an initial response rate of 83.2% for HCWs from OAHs.

Of the 360 questionnaires distributed at the study sites, 287 were returned and of these, 11 questionnaires had to be removed due to missing key data (e.g.: the question on “ever received influenza vaccination” was not answered) and/or inconsistencies (e.g.: responding “no” to ever having received an influenza vaccine, but then giving a reason for receiving the vaccine) in the responses. A final response rate of 76.6% (276/360) for fully completed questionnaires with consistent data was therefore obtained. The final HCW response rates for CHCs and OAHs were 69.9% (165/236) and 89.5% (111/124) respectively.

### Socio-demographic characteristics

Females constituted 90.9% (251/276) of the participants. The majority of participants were Black African (77.5% [214/276]), and the mean age was 41.1 years (SD: 11.7; range: 19.3-68.5). The highest proportion of participants were in the age group 30-34 years (18.1% [50/276] with the majority of participants (62.7% [173/276]) being younger than 45 years. Nurses accounted for the greatest proportion (82.7% [143/276]) of participants and participants were mostly employed in CHCs (59.8% [165/276]). The mean number of years of clinical experience was 11.0 years (SD: 9.1; range: 0.2-43.0), with 72.8% having less than 15 years’ experience.

### Influenza vaccine uptake and recommendation of influenza vaccination to patients

Overall, 62.7% (173/276) of participants indicated having ever received at least one dose of the influenza vaccine with 24.3% (41/276) and 33.3% (92/276) indicating having received the vaccine in 2017 and 2018, respectively. Table 3 gives information on HCW influenza vaccine uptake, stratified by socio-demographic characteristics of participants. Of those ever receiving an influenza vaccination, 2.3% (4/173) were vaccinated only in 2018; 50.9% (88/173) were vaccinated in 2018 and prior to 2018; and 46.8% (81/173) were vaccinated prior to 2018, but not in 2018 (See Table 4). Of those vaccinated in 2018 and prior to 2018, 97.7% (86/88) had received previous influenza vaccinations in the past 3 years: 27.3% (24/88) in 2017; 3.8% (35/88) in 2016; and 30.7% (27/88) in 2015. Most HCWs (67.8% [187/276]) indicated that they recommend influenza vaccines to elderly patients in their care.

**Table 3:** Socio-demographics of HCWs stratified by history of ever receiving seasonal influenza vaccination (*n* = 276)

Variable	Ever vaccinated	Never vaccinated	Totals
	***n*** (%)	***n*** (%)	***n*** (%)
**Profession**			
Nurses	143 (63.8)	81 (36.2)	224 (81.2)
* Registered nurse*	*65 (73.0)*	*24 (27.0)*	*89 (32.2)*
* Auxiliary nurse*	*47 (58.0)*	*34 (42.0)*	*81 (29.3)*
* Enrolled/staff nurse*	*31 (57.4)*	*23 (42.6)*	*54 (19.6)*
Pharmacists	8 (61.5)	5 (38.5)	13 (4.7)
Pharmacy assistants	8 (72.7)	3 (27.3)	11 (4.0)
Medical practitioners	5 (45.5)	6 (54.5)	11 (4.0)
Dental practitioner	4 (44.4)	5 (55.6)	9 (3.3)
Other^a^	5 (62.5)	3 (37.5)	8 (2.9)
**Sex**			
Male	15 (60.0)	10 (40.0)	25 (9.1)
Female	158 (62.9)	93 (37.1)	251 (90.9)
**Race**			
Black	138 (64.5)	76 (35.5)	214 (77.5)
Coloured	18 (48.6)	19 (51.4)	37 (13.4)
White	16 (76.2)	5 (23.8)	21 (7.6)
Indian	1 (50.0)	1 (50.0)	2 (0.7)
Other	0 (0.0)	2 (100.0)	2 (0.7)
**Age group in years**			
18–20	0 (0.0)	1 (100.0)	1 (0.4)
20–24	11 (57.9)	8 (42.1)	19 (6.9)
25–29	20 (58.8)	14 (41.2)	34 (12.3)
30–34	29 (58.0)	21(42.0)	50 (18.1)
35–39	23 (67.6)	11 (32.4)	34 (12.3)
40–44	25 (71.4)	10 (28.6)	35 (12.7)
45–49	16 (57.1)	12 (42.9)	28 (10.1)
50–54	22 (61.1)	14 (38.9)	36 (13.0)
55–59	14 (70.0)	6 (30.0)	20 (7.2)
60–64	11 (84.6)	2 (15.4)	13 (4.7)
> 64	2 (33.3)	4 (66.7)	6 (2.2)
**Work experience in years**			
< 5	40 (49.4)	41 (50.6)	81 (29.3)
5–9	38 (53.5)	33 (46.5)	71 (25.7)
10–14	38 (77.6)	11 (22.4)	49 (17.8)
15–19	21 (77.8)	6 (22.2)	27 (9.8)
20–24	13 (68.4)	6 (31.6)	19 (6.9)
25–29	11 (78.6)	3 (21.4)	14 (5.1)
30–34	7 (77.8)	2 (22.2)	9 (3.3)
35–39	2 (100.0)	0 (0.0)	2 (0.7)
> 39	3 (75.0)	1 (25.0)	4 (1.4)
**Sector**			
Community Health Centre	99 (60.0)	66 (40.0)	165 (59.8)
Old Age Home (Private)	38 (62.3)	23 (37.7)	61 (22.1)
Old age Home (NGO)	36 (72.0)	14 (28.0)	50 (18.1)
**Totals**	**173 (62.7)**	**103 (37.3)**	**276 (100)**

*HCWs* Healthcare workers, *NGO* Non-governmental organizations

^a^’Other’ includes qualified and student allied HCWs such as physiotherapists, social workers and dieticians

**Table 4:** Frequency distribution for the year of influenza vaccination (*n* = 169)

Year of last influenza vaccination prior to 2018	***n*** (%)
**2017**	41 (24.3)
**2016**	88 (52.1)
**2015**	33 (19.5)
**2014**	2 (1.2)
**2013**	2 (1.2)
**2012**	1 (0.6)
**2008**	2 (1.2)

### Reasons for vaccination status

The most common reason for accepting influenza vaccination was to prevent illness (protect themselves) (91.3% [84/92] in 2018 and 94.1% [159/169] for prior influenza vaccination). Reasons related to the unavailability of the influenza vaccine (i.e. no stock or not being offered the vaccine) at the facilities, was the most common category of reasons for not receiving the vaccine, reported by 66.8% (123/184) of those not vaccinated in 2018, and 70.9% (73/103) of those who had never been vaccinated. Reasons related to vaccine hesitancy (i.e. “Experienced bad side effects with vaccines” or “Lack of faith in the influenza vaccine”) was the second most common category of reasons for not receiving the vaccine, reported by 31.0% (57/184) of those not vaccinated in 2018, and 27.2% (28/103) of those who had never been vaccinated. Detailed information on the reasons for receiving the influenza vaccine or not receiving the influenza vaccine by the HCWs in 2018 and prior to 2018 are presented in Tables 5 and 6 respectively.

**Table 5:** Frequency distribution for reasons for accepting influenza vaccination

	Prior to 2018 (***n*** = 169)	2018 (***n*** = 92)
**Reasons for accepting influenza vaccination**	***n*** **(%)**	***n*** **(%)**
To protect themselves against influenza	159 (94.1)	84 (91.3)
To minimise transmission of influenza to patients	6 (3.6)	7 (7.6)
To obtain Vitality Points^b^	3 (1.8)	1 (1.1)
Encouraged to receive vaccine by their own healthcare provider	1 (0.6)	0 (0.0)

^b^Vitality Points are rewards from a medical insurance loyalty programme awarded to the beneficiary after they complete educational, fitness, healthy living and/or preventative activities toward the achievement of wellness goals

**Table 6:** Frequency distribution of reasons for not receiving influenza vaccination

Reasons	*Not vaccinated before 2018 (***n*** = 107)	*Not vaccinated in 2018 (***n*** = 184)	Vaccinated before 2018, but not in 2018 ***(n*** = 81)	Never vaccinated (***n*** = 103)
	***n*** (%)	***n*** (%)	***n*** (%)	n (%)
Experienced bad side effects with vaccines	4 (3.7)	21 (11.4)	17 (21.0)	4 (3.9)
Forgot to receive the influenza vaccine	15 (14.0)	3 (1.6)	2 (2.5)	1 (1.0)
No vaccine stock available	72 (67.3)	78 (42.4)	13 (16.0)	65 (63.1)
Influenza vaccine not offered by employer or own healthcare provider	5 (4.7)	45 (24.5)	37 (45.7)	8 (7.8)
Lack of faith in the vaccine	9 (8.4)	36 (19.6)	12 (14.8)	24 (23.3)
Did not know about the influenza vaccine	2 (1.9)	1 (0.5)	0 (0.0)	1 (1.0)

*Includes some of those in the ‘Never vaccinated’ column

## Discussion

We believe this is the first study undertaken in South Africa to assess influenza vaccination coverage among HCWs caring for the elderly, a vulnerable population at high risk for severe outcomes from influenza. The low influenza vaccination coverage amongst HCWs reported in this study, is supported by results from previous South African studies [11, 39–41, 51], ranging from 21% in a Gauteng tertiary hospital [41], to 34% in HCWs from 21 districts in 5 provinces [39]. Nevertheless, there was a marked improvement in vaccine uptake in 2018 (33.3%) when compared to 2017 (24.3%). However, when taking into account that of those ever receiving influenza vaccination, only 2.3% had received their first vaccine in 2018, while 46.8% were not vaccinated in 2018, this apparent “improvement” does not reflect an overall improvement in the influenza vaccination programme for HCWs employed in South Africa.

South Africa is one of many low- and middle-income countries (LMICs) with sub-optimal influenza vaccination coverage of HCWs [52]. A 2020 survey completed by 68 LMICs, including South Africa, found that only 51.5% had an influenza vaccination policy for HCWs, mainly because influenza was not prioritised in countries without a policy. Only 22 LMICs provided coverage rates for HCWs for the previous influenza season, ranging from 0% to 100%, with a mean of 57%, and with only seven countries having achieved the HCW coverage target of 75% [52].

Low influenza vaccination coverage in HCWs is not confined to LMICs. It is a global phenomenon seen in many high-income countries (HICs) as well [26, 32, 33, 53–55]. This is despite the existence of guidelines and recommendations that promote vaccination of HCWs in these countries [56, 57]. Most countries in Europe have an influenza vaccine uptake rate lower than 40%, with only three countries achieving the European HCW coverage target of 75% in the period 2008/2009 to 2014/2015 [56–58]. Similarly, influenza vaccine uptake amongst HCWs in six major hospitals in Saudi Arabia during the 2012–2013 season was only 38% [32]. In contrast, the majority of healthcare facilities in the United States have attained much higher influenza vaccination coverage by mandating influenza vaccination for HCWs, with 96.7% coverage attained in healthcare facilities where influenza vaccination was mandated [57]. These vaccine mandates are supported by providing HCW education about influenza vaccination and easily accessible free influenza vaccination services [57]. There are several possible explanations for low vaccination coverage rates for HCWs in HICs other than the United States. These include: the perception that influenza prevention has low importance due to the absence of vaccination policies; a decline in vaccine confidence; lack of funding for HCW influenza vaccination programmes; inconsistent methods for estimating influenza vaccine coverage in some countries; and differing regulatory environments for HCW vaccination [52, 59].

Our finding that influenza vaccine uptake rates were highest amongst registered nurses, and lowest amongst medical and dental practitioners, is supported by other studies from South Africa [39, 41, 51]. In contrast, studies from HICs have found higher rates among medical practitioners. For example, an analysis of influenza vaccine uptake rates by HCWs in the United States during the 2017-2018 influenza season, found the highest uptake rate amongst medical practitioners (96.1%), with 90.5% of registered nurses being vaccinated, while the lowest vaccine uptake rates were found amongst non-medical staff [13]. Similarly, a study from Italy found the highest vaccination rates in medical practitioners, and furthermore, being a medical practitioner was a statistically significant predictor of influenza vaccination uptake [60]. In addition, a study from Ireland that analysed influenza vaccination uptake by HCWs employed in public hospitals and long-term or residential care facilities (which includes OAHs) from 2011 to 2020, consistently reported much higher uptake by medical practitioners and dentists working in public hospitals, than nurses. However, among HCWs employed at long-term or residential care facilities, uptake was occasionally (in 2011-2012 and 2017-2018) slightly higher in nurses, and although medical practitioners had higher vaccination uptake in all other years, the difference in uptake between the two professions was not as great as the difference in those employed at public hospitals [61]. These differences may be explained by differing levels of vaccine hesitancy and perceptions regarding the severity of influenza illness among the different HCW professions [59, 60, 62].

In our study, HCWs working in CHCs had a lower influenza vaccination coverage than those working in OAHs, where HCWs specifically take care of the elderly. In contrast, studies conducted in HICs have found lower influenza vaccination coverage in HCWs employed at long-term or residential care facilities, than in other healthcare facilities [13, 61]. A survey conducted in the United States linked this lower coverage to the absence of facility-based influenza vaccination promotion, with no policy requiring influenza vaccination for HCWs, in addition to not providing HCWs with free and easy access to the vaccine [13]. Thus our finding of a higher coverage in HCWs working in OAHs suggests that vaccine advocacy may be taking place, and some OAHs may be providing free and easy access to the vaccine, or may have made vaccination a requirement for employment. A possible reason for the higher coverage could be that HCWs in OAHs may have private medical insurance and many of these medical insurance schemes promote influenza vaccination with full coverage and not from members' savings accounts). Based on our findings, we are planning to investigate the vaccination policies of OAHs in future research projects.

In South Africa, in order to protect the most vulnerable and mitigate the impact of absenteeism during the COVID-19 pandemic, the NDoH prioritised HCWs for free influenza vaccination for the 2020 and 2021 influenza seasons [21, 38]. In addition, following the national roll-out of the COVID-19 vaccination programme in early 2021, the NDoH has strongly promoted both COVID-19 vaccination and influenza vaccination for HCWs and the elderly, which is the current recommendation [21, 31, 63]. It can thus be anticipated that there may be an improvement in influenza vaccination uptake in all HCWs [57, 64, 65]. Since the elderly are especially vulnerable to both influenza and COVID-19, we are expecting to see a vast improvement in influenza vaccination uptake in HCWs employed at CHCs and OAHs, and will be following-up on this in the future.

The main reason for receiving influenza vaccination among HCWs in this study, was to protect themselves, with a lower proportion vaccinating themselves in order to protect their patients. This finding is in agreement with other studies conducted in South Africa [41, 51] and globally. For example, a 2018-19 study carried out in the Netherlands [58] found that the most frequently reported motives for vaccinating were personal protection against influenza and lowering of the risk of transmitting influenza to patients. A systematic review that investigated vaccination amongst HCWs also found that the main driver for vaccine uptake was a desire to protect themselves and their families [33]. The risk of illness and complications due to influenza were less common reasons for vaccination [33].

Our study identified the unavailability of the influenza vaccine as the principal reason for non-vaccination. While most South African studies have reported vaccine unavailability as one of the reasons for not being vaccinated, this has not always been the major reason. The complexity of access as a predictor of vaccination uptake in the South African setting, is well illustrated by a previous study conducted in five South African provinces, where only 34% of 1164 HCWs had received influenza vaccination in the previous influenza season [39]. In answer to influenza vaccination access-related questions, 81% reported having access, with 65% having access to free vaccination, while only 49% reported having access to influenza vaccination services at their workplace. Furthermore, statistically significant positive associations were found between vaccine uptake and access to the vaccine (odds ratio [OR]: 3.52) and receiving the vaccine free of charge (OR: 1.67). However, the availability of the influenza vaccine in the workplace was not identified as a predictor of vaccine uptake [39]. Notwithstanding these results, the provision of free influenza vaccination services during working hours at the facilities where HCWs are employed, has been shown to be a successful strategy that is highly recommended for all LMICs [52]. The results of our study show how important this strategy is, since 46% of HCWs who were vaccinated in previous years but not in 2018, cited not being offered the vaccine by their employer or own healthcare provider, as the reason for not being vaccinated.

Reasons related to vaccine hesitancy ranked second amongst all reasons for non-vaccination in this study. Vaccine hesitancy has previously been identified as one of the barriers to uptake of influenza vaccination by South African HCWs, but its importance has varied between facility type and geographical location [41, 51]. For example, a survey conducted at a tertiary hospital in Gauteng Province in 2019 (where only 21% of 412 HCWs had received influenza vaccination in the previous influenza season), reported that 66% of unvaccinated HCWs cited reasons related to vaccine hesitancy [41]. In contrast, a survey conducted at 30 primary healthcare clinics in North West Province in 2018 (where only 29% of 272 HCWs had received influenza vaccination in the previous influenza season), reported that 21% of unvaccinated HCWs cited reasons related to vaccine hesitancy [51]. Of interest, our study found that those vaccinated before 2018 but not in 2018, reported relatively high levels of reasons related to vaccine hesitancy, with 21% reporting bad side effects, and 15% reporting lack of faith in the influenza vaccine. This suggests that these HCWs may have contracted a respiratory infection caused by one of the many viruses circulating during the influenza season when they were last vaccinated. If this infection coincidentally happened shortly after receiving the vaccine, the vaccine itself may have been blamed for causing the infection (hence the fear of “bad side effects”), while if it happened later on in the season, the effectiveness of the vaccine may have been questioned (hence “lack of faith”). These misconceptions point to the need for training of HCWs employed at these health facilities, since the receipt of influenza training provided by the NDoH has been identified as a statistically significant predictor of influenza vaccination uptake [39]. Vaccine hesitancy resulting from misconceptions rooted in a lack of knowledge about influenza and influenza vaccines, has been identified as a major barrier to uptake by HCWs throughout the world [33], in both HICs [12, 32, 59, 60] and LMICs [34, 52, 66].

Despite the very low influenza vaccination coverage of HCWs participating in this study, 68% of participants recommended influenza vaccination to their elderly patients. In contrast, a previous study conducted amongst HCWs in five provinces of South Africa, reported that 94% of participants recommended influenza vaccination to their patients (in general, and to their HIV-positive patients in particular) [39]. In addition, logistic regression analysis identified having received influenza vaccination themselves in the previous season, and the availability of the influenza vaccine during the healthcare visit, as statistically significant predictors of them recommending influenza vaccination to their HIV-positive patients [39]. Bearing in mind that both these studies reported low influenza vaccination coverage amongst participants, the differences between the two studies in (a) availability of the vaccine and (b) perceptions of the risk of influenza to their patients, are likely to have made major contributions to the different rates of recommendations to their patients. Patients rely on HCWs as a trusted source of health advice, and vaccine uptake amongst patients is positively associated with the perception of the vaccine and recommendations by their own healthcare provider [35, 66–68]. In addition, HCWs may help to address any misinformation about the influenza vaccine and respond to any concerns of vaccine hesitant patients [67, 68]. Several studies have shown that high levels of knowledge about and positive attitudes towards influenza vaccination, are positively associated with high uptake of the vaccine by HCWs [26, 27, 50, 65, 69–71]. Furthermore, vaccinated HCWs have a greater intention to vaccinate patients under their care [26, 70].

### Study limitations

This study was limited to descriptive statistical analysis, as it was not sufficiently powered for inferential statistical analysis and sub-group analysis. A larger sample size would have allowed for determining associations and identifying predictors or barriers. Furthermore, since nursing professionals constitute the major HCW cadre in the South African setting, especially in OAHs where only 4% are visited by a doctor on a daily basis [45], the vast majority of participants in this study were nurses. Thus future studies should include diverse settings where more medical practitioners with high numbers of elderly patients can be surveyed, such as geriatricians and medical specialists focused on treating health problems disproportionately affecting the elderly.

In addition, the research objectives addressed by this survey did not require the collection of data on participants’ health status, including any co-morbidities. Thus HCWs with underlying chronic conditions such as HIV infection for which the influenza vaccine is prioritised by the NDoH guidelines, could not be identified and analysed as a separate target group.

Another limitation is that a convenience sample of the study sites was used. Also, the study was limited to CHCs and private sector OAHs, thus HCWs who work in public sector OAHs and other healthcare facilities such as hospitals, were not represented in the study population. Furthermore, although no sampling was conducted at health-facility level, the study is subject to volunteer bias. Thus the results of the study are not necessarily representative and therefore not generalisable to all HCWs caring for the elderly in the South Africa setting.

Surveys on vaccination coverage of HCWs are necessary in South Africa, because there is no centralised “whole of life” immunisation registry, and facility-based records of HCW vaccinations are generally not available. Thus, data on vaccine uptake and reasons for non-vaccination collected for this study were self-reported, and are therefore subject to recall bias.

Finally, non-medical workers such as cleaning staff, administrative staff and catering staff were not surveyed in this study. These workers are also sources of transmission of influenza to other HCWs and vulnerable elderly persons at CHCs or OAHs. There is thus a gap in these data, which needs to be addressed by future studies.

Despite the limitations, this was a nationwide study conducted in all nine provinces of South Africa, and in the major healthcare access points utilised by the elderly. This allows for some degree of generalisability of the study results to HCWs in South Africa who frequently interact with and take care of the elderly. Furthermore, the findings can be used to guide the development of future policies and strategies in South Africa to further protect the elderly, and as a baseline to measure the impact of these policies and strategies.

## Conclusions and recommendations

Whilst higher than preceding years, this study found low influenza vaccination uptake amongst HCWs caring for the elderly in 2018, despite published NDoH recommendations and guidelines that encourage influenza vaccination of HCWs. Unavailability of the influenza vaccine and reasons related to vaccine hesitancy were the main reasons for low uptake. In addition, many of the participants in this study were not recommending influenza vaccination to their elderly patients, despite NDoH guidelines prioritizing the elderly for receiving free influenza vaccination. Following the advent of the COVID-19 pandemic, priority interventions such as providing free influenza vaccination and increasing the awareness of HCWs on the risks of transmitting influenza to patients in their care, were implemented by the NDoH. It now remains to be seen if these interventions have been successful, and we will be using the data from this study as a baseline to compare the results of a future survey, which is currently in the pipeline.

Alongside this, South African higher education institutions that train HCWs must review their nursing and medical curricula, and ensure that pre-service and in-service training on the risks associated with influenza illnesses and associated risks are highlighted for all HCWs. In addition, the training should emphasise the safety, effectiveness and importance of influenza vaccination and address issues of vaccine hesitancy. Implementation of the updated NDoH influenza guidelines providing free influenza vaccination to both HCWs and the elderly, combined with interventions to increase HCW influenza vaccination coverage and translating the increased knowledge of the risks of influenza and benefits of influenza vaccination for the elderly into practice, should enhance compliance with these guidelines, and safeguard the health of the elderly. This is particularly important as South Africa moves towards achieving universal health coverage, since adequate training on the importance of HCW vaccination alongside the availability of vaccines free-of-charge for HCWs and the elderly, are essential for minimizing the risks associated with nosocomial infections in this vulnerable group.

## Supplementary Information

**Additional file 1.** Participant questionnaire (HCWs).

**Additional file 2.** Figure S1 Planned study sites and participant sampling process.

## Data Availability

The data generated from this study are available on request to the corresponding authors.
