# Supplementary material for: Low influenza vaccine uptake by healthcare workers caring for the elderly in South African old age homes and primary healthcare facilities
Source: BMC Public Health. 2023 Jan 12;23:91. doi: 10.1186/s12889-022-14926-8 (PMC9834679; doi:10.1186/s12889-022-14926-8)
Supplement: Supplementary file 1 — Additional file 1. Participant questionnaire (HCWs). [file 12889_2022_14926_MOESM1_ESM.docx]

Participant questionnaire (HCWs)

| **Date:** |  | **Province:** | |  |
| --- | --- | --- | --- | --- |
| **Participants Study Number:** |  | **Facility Study Number:** | |  |
| **Gender** | Male |  | Female |  |
| **Date of birth** |  | | | |
| **Qualification** | Doctor |  | Professional nurse |  |
|  | Pharmacist |  | Enrolled/staff nurse |  |
|  | Pharmacist assistant |  | Other (Specify) |  |
| **Ethnic Group** | African |  | Indian |  |
|  | Coloured |  | Other Asian |  |
|  | White |  | Other (Specify) |  |
| **Employment Sector** | CHC |  | OAH (NGO) |  |
|  | OAH (Public) |  | OAH (Private) |  |
| **Years of work experience** |  | | | |

**Vaccination status (Mark with “X” on the corresponding answer)**

| **Vaccine** | **Vaccination status** | | | | | **Reason/s why I did / did not receive the vaccine** |
| --- | --- | --- | --- | --- | --- | --- |
| **Have you ever been vaccinated against Influenza?** | Yes |  | **Year/s?** |  | |  |
|  | No |  | | | |  |
|  | Don’t know |  | | | |  |
| **Were you vaccinated against influenza this year? (2017/2018)** | Yes |  | | | |  |
|  | No |  | | | |  |
|  | Don’t know |  | | | |  |
| **Are you vaccinated against Hepatitis B?** | Yes |  | **Year/s** | |  |  |
|  | No |  | | | |  |
|  | Don’t know |  | | | |  |
| **If you were vaccinated against Hepatitis B, How many doses did you receive?** | | | | | |  |
| **Do you routinely recommend vaccines to elderly patients in your care?** | Yes |  | | | |  |
|  | No |  | | | |  |
| **When you were a child, did you receive all the recommended vaccines?** | Yes |  | | | |  |
|  | No |  | | | |  |
|  | don’t know |  | | | |  |
| **If you did not receive all the recommended childhood vaccines, have you since received some of these vaccines as an adult?** | Yes |  | **Year/s?** | |  |  |
|  | No |  | | | |  |
|  | Don’t know |  | | | |  |
| **If you did not receive all the recommended childhood vaccines as a child but have since received some of these vaccines as an adult, specify the vaccines you received?** | | | | | |  |

**Are you willing to receive vaccine updates via your mobile phone? If yes, please give the reasons for your answer. If No, please explain why not.**

**Are you willing to participate in a follow up focus group discussion or in-depth interview? If yes, please supply a contact telephone number for follow up. If no, leave blank.**
