# Supplementary material for: Low influenza vaccine uptake by healthcare workers caring for the elderly in South African old age homes and primary healthcare facilities
Source: BMC Public Health. 2023 Jan 12;23:91. doi: 10.1186/s12889-022-14926-8 (PMC9834679; doi:10.1186/s12889-022-14926-8)
Supplement: Supplementary file 2 — Additional file 2. Figure S1 Planned study sites and participant sampling process. [file 12889_2022_14926_MOESM2_ESM.docx]

**Figure S1: Planned study sites and participant sampling process**

|  | **18 Community Healthcare Centres (CHCs)**  **(2 per Province)** | **27 Old Age Homes (OAHs)**  **(3 per Province)** |
| --- | --- | --- |
| **STUDY SITES** |  | **9 Public**    **9 NGO**    **9 Private** |
| **SAMPLE** | **11 x HCWs per CHCs**  **(n=198)** | **6 x HCWs per OAH**  **(n=54)**  **6 x HCWs per OAH**  **(n=54)**  **6 x HCWs per OAH**  **(n=54)** |
|  |  |  |
|  |  | **Healthcare Workers (HCWs) (N = 360)** |
